# Baseline Differences in Anxiety Affect Attention and tDCS-Mediated Learning

**DOI:** 10.3389/fnhum.2021.541369

**Published:** 2021-03-03

**Authors:** Benjamin C. Gibson, Melissa Heinrich, Teagan S. Mullins, Alfred B. Yu, Jeffrey T. Hansberger, Vincent P. Clark

**Affiliations:** ^1^Department of Psychology, Psychology Clinical Neuroscience Center, University of New Mexico, Albuquerque, NM, United States; ^2^The Mind Research Network of the Lovelace Biomedical Research Institute, University of New Mexico, Albuquerque, NM, United States; ^3^DEVCOM Army Research Laboratory, Human Research, and Engineering Directorate, Aberdeen Proving Ground, MD, United States

**Keywords:** tDCS, brain stimulation, attention, learning, anxiety, individual differences, perception, visual learning

## Abstract

Variable responses to transcranial direct current stimulation (tDCS) protocols across individuals are widely reported, but the reasons behind this variation are unclear. This includes tDCS protocols meant to improve attention. Attentional control is impacted by top-down and bottom-up processes, and this relationship is affected by state characteristics such as anxiety. According to Attentional Control Theory, anxiety biases attention towards bottom-up and stimulus-driven processing. The goal of this study was to explore the extent to which differences in state anxiety and related measures affect visual attention and category learning, both with and without the influence of tDCS. Using discovery learning, participants were trained to classify pictures of European streets into two categories while receiving 30 min of 2.0 mA anodal, cathodal, or sham tDCS over the rVLPFC. The pictures were classifiable according to two separate rules, one stimulus and one hypothesis-driven. The Remote Associates Test (RAT), Profile of Mood States, and Attention Networks Task (ANT) were used to understand the effects of individual differences at baseline on subsequent tDCS-mediated learning. Multinomial logistic regression was fit to predict rule learning based on the baseline measures, with subjects classified according to whether they used the stimulus-driven or hypothesis-driven rule to classify the pictures. The overall model showed a classification accuracy of 74.1%. The type of tDCS stimulation applied, attentional orienting score, and self-reported mood were significant predictors of different categories of rule learning. These results indicate that anxiety can influence the quality of subjects’ attention at the onset of the task and that these attentional differences can influence tDCS-mediated category learning during the rapid assessment of visual scenes. These findings have implications for understanding the complex interactions that give rise to the variability in response to tDCS.

## Introduction

Attentional control is assumed to occur through bottom-up, implicit processes and top-down, consciously instigated processes, with this distinction codified *via* behavioral (Jonides, 1981; Wolfe et al., 1989) and neuroscience measures (Desimone and Duncan, 1995; Corbetta and Shulman, 2002; Mazaheri et al., 2011). While the usefulness of this distinction has been called into question, and processes outside this dichotomy have been proposed (Awh et al., 2012; Theeuwes, 2018), top-down and bottom-up attention remain the theoretical cornerstones of many cognitive models of attention and visual search (Posner, 1978; Duncan and Humphreys, 1989; Wolfe et al., 1989; Found and Müller, 1996; Ludwig and Gilchrist, 2002; Serences and Boynton, 2007). Part of the reason for their ubiquity is that this dichotomy provides ample room for interpretation (Wolfe et al., 2003; Lien et al., 2010; Theeuwes, 2010, 2018). Top-down and bottom-up represent broad directions for information flow that exist along an overlapping gradient. There is no discrete starting point for attention defined as top-down or bottom-up; rather, an attentional event begins somewhere along this gradient as dependent upon a host of state or trait influences (Katsuki and Constantinidis, 2014).

One factor known to bias attention towards implicit processing is anxiety (Bishop, 2007). Anxiety is often found to be detrimental to cognitive performance, with performance declining as complexity and attentional demands increase (Hembree, 1988; Eysenck and Calvo, 1992; Orem et al., 2008; Derakshan and Eysenck, 2009; Moran, 2016). Attentional Control Theory provides an account of how anxiety impacts attention and negatively impacts higher-level cognitive processing (Eysenck et al., 2007). This theory proposes that there are two competing systems of attention; a purpose-driven, top-down system, and a stimulus-driven, bottom-up system. Anxiety alters the balance of these competing systems in favor of bottom-up processing (Derryberry and Reed, 2002; Eysenck et al., 2007). This dichotomy is supported by neuroimaging, which has found partially overlapping substrates for these two systems (Corbetta and Shulman, 2002; Corbetta et al., 2008).

The antisaccade task has served as a behavioral analog for measuring changes in the balance of the competing systems in Attentional Control Theory (Miyake et al., 2000; Derakshan et al., 2009). In the antisaccade task, subjects are required to inhibit a reflexive saccade towards a sudden visual stimulus presented in the periphery and instead generate a purposeful saccade in the opposite direction. Purposeful and automatic saccades thus compete, with anxiety serving to suppress the former (Hunt et al., 2004; Massen, 2004). Administration of 7.5% CO_2_ has been used as a temporary way of increasing self-report anxiety and modeling generalized anxiety disorder in healthy volunteers (Bailey et al., 2007, 2011). Subjects given 7.5% CO_2_ have demonstrated a decreased ability to purposefully control eye movements in the antisaccade task (Garner et al., 2011). This ability is linked to the orienting network, which directs attention through space and is distinct from two other attention networks, executive and alerting. Appropriately, subjects given 7.5% CO_2_ exhibit greater orienting scores on the Attention Networks Task (ANT; Fan et al., 2002), demonstrating that this measure provides a way of quantifying attentional changes resulting from anxiety.

In viewing naturalistic scenes, the role of explicit attention is emphasized, where it has been proposed that attention falls more readily on pertinent rather than salient objects (Castelhano and Henderson, 2007; Henderson et al., 2009), with pertinence determined by explicit goals (Torralba et al., 2006; Neider and Zelinsky, 2008; Ehinger et al., 2009). However, while the factors that drive attention during Freeview of natural scenes have been described (Wolfe and Horowitz, 2017), the influence of baseline differences on these factors is less understood, especially in situations where preconscious knowledge and explicit goals are lacking. The trajectory of attention is then either driven by explicit ad-hoc goals, or by baseline differences, such as anxiety, that serve to influence the salience of items in the visual field (Itti and Koch, 2000; Wolfe and Horowitz, 2017). To explore these possibilities, the present study created a novel learning task where naturalistic stimuli could be categorized according to either top-down, hypothesis-driven, or bottom-up, stimulus-driven rules.

This study additionally employed transcranial direct current stimulation (tDCS), and the main effect of tDCS on learning is reported elsewhere (Gibson et al., 2020). Over the past several decades, tDCS, a form of non-invasive brain stimulation, has been coupled with behavioral interventions in an attempt to improve their efficacy (Clark and Parasuraman, 2014; Coffman et al., 2014). This includes functions classified under the umbrella of attention, with stimulation having been applied to areas across the cortex to try and improve different aspects of attention (Reteig et al., 2017). Some tDCS studies have found success in improving sustained attention by stimulating the right ventrolateral prefrontal cortex (rVLPFC; Clark et al., 2012; Coffman et al., 2012; Falcone et al., 2012; Nelson et al., 2014), a finding that is consistent with the association of the rVLPFC with the maintenance of attention and cognitive control (Coull et al., 1998, 2001; Aron et al., 2003, 2014; Hampshire et al., 2009, 2010). Besides attention, the rVLPFC is associated with other cognitive processes like convergent creativity, hypothesis testing, and rule learning (Seger et al., 2000; Bowden and Jung-Beeman, 2003a; Jung-Beeman et al., 2004; Seger and Cincotta, 2006; Goel et al., 2007; Mashal et al., 2007; Mihov et al., 2010; Crescentini et al., 2011; Cao et al., 2016). To examine how baseline differences in these constructs, as well as anxiety, influence tDCS-mediated category learning, subjects were tested on measures of convergent creativity, attention, and self-report state affect, before application of tDCS. The degree of interaction between these baseline measures and learning performance was then quantified. It was hypothesized that higher baseline anxiety would bias subjects to learn the stimulus-driven rather than hypothesis-driven rule.

## Materials and Methods

### Subjects

Subjects were recruited through the research portal of the University of New Mexico (UNM) and advertisements posted in and around the UNM campus. Subjects received either cash payment (approximately $30) or class credit for a single experimental visit that lasted around 2 h. Before enrollment, subjects were screened for typical tDCS inclusion criteria (Bikson et al., 2016). At the beginning of the experimental session, subjects were informed of the details and goals of the study, including the use of tDCS, and consented. Study materials and procedures were approved by the U.S. Army Research Laboratory’s Human Research Protection Program and by Chesapeake IRB.

### Experimental Task

**Figure 1:**
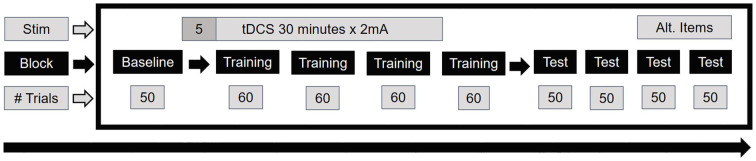
Timing of stimulation and design of the main task. A total of 54 subjects were run, with 18 each in the anodal, cathodal, and sham conditions.

A novel paradigm using naturalistic visual stimuli was created for this study, where subjects were tasked with learning to classify pictures of European streets as belonging to one of two categories. These categories were associated with buttons 1 and 2 on the keyboard number pad. The stimuli were static street segment views accessed on Google Maps Street View^1^. During each trial a single static street view was presented for 2.5 s, followed by a fixation cross for 1.5 s. After an initial baseline block of 50 trials, there was a training portion consisting of four blocks of 60 trials each, where after each trial subjects received feedback on their classification accuracy. Feedback consisted of screens reading “Correct” or “Incorrect,” simultaneously accompanied by male voices with European accents reciting a range of feedback congruent with the written feedback. Following training, there were four test blocks of 50 trials each, all without feedback (Figure 1). The baseline block was framed as practice during which subjects were instructed to become accustomed to the timing of the stimuli and to begin thinking about criteria that might differentiate the two categories. Subjects were told that there were two regions but were not given any clues about possible ways to distinguish them, nor were they told that there might be more than one way to differentiate the regions. Instead, through discovery learning (Bruner, 1961), they were tasked with gleaning the identifying features *via* accuracy feedback in the training portion. The instruction screen read, “This task uses “discovery learning,” meaning that you do not know what separates one category from another at the start. All you know is that two categories exist. You will learn what separates the categories during feedback in the training portion. Any number of features could be critical to separating one category from another.”

**Figure 2:**
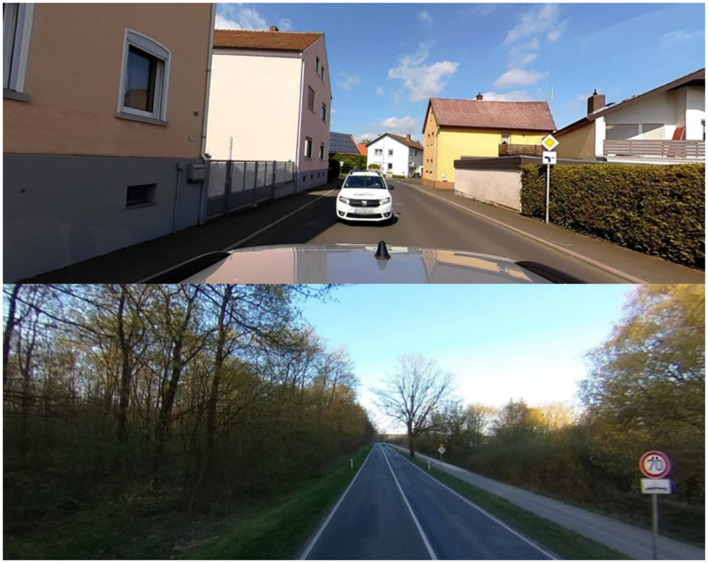
Example of rule 1 stimuli with approaching traffic (top picture) and rule 2 stimuli (bottom picture) with an umlaut (located on speed limit sign).

**Figure 3:**
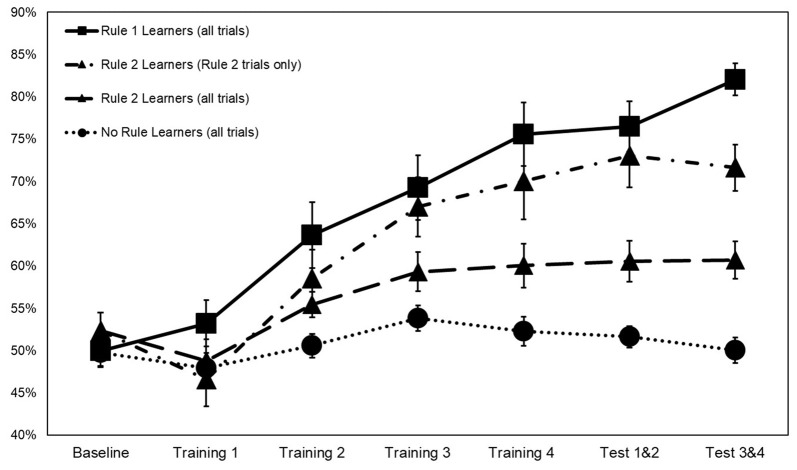
Categorization accuracy by rule group across training with rule 2 learners represented both on rule 2 accuracy only and on overall accuracy. Error bars ±1 SE.

Pictures could be correctly categorized through two arbitrary rules. The first rule differentiated regions based on how the picture was taken in relation to the road. In region 1, pictures were taken on the left-hand side of the road with traffic approaching, while in region 2, pictures were taken on the right-hand side of the road with traffic moving away (rule 1; Figure 2). The traffic pattern was on the right across all pictures. Rule 1 was created to model a bias towards explicit attention, as success in deciphering this rule does not depend on orienting towards items in the pictures, but rather gleaning the insight that rule 1 depends on the way the picture itself is taken. The second rule consisted of symbols within the pictures (i.e., hidden objects). Two side-by-side dots (umlaut) were in pictures for region 1, and a curved line (tilde) was in the pictures in region 2 (rule 2; Figure 3). The size of the two objects was standardized in pixels by height and width. Rule 2 was created to model a bias towards implicit attention driven by an orienting response. Hidden objects were only placed on human artifacts to accord with likely patterns of syntactic and semantic guidance (Biederman et al., 1982). Within the baseline, training, and first 2 test blocks, rule 1 (street direction) was present in all trials, while rule 2 (hidden objects) was present in 50% of the trials within each block. The last two test blocks contained trials designed to isolate and test learning of each rule individually. This consisted of 50 repeat images (all hidden object trials from the training portion where the hidden object had been removed) to preferentially isolate learning of rule 1, and 50 hidden object trials to preferentially isolate learning of rule 2 (where the street direction previously associated with each of the hidden objects was reversed). To ensure consistency throughout the task, the conspicuousness of each of the rules in individual pictures was rated on a 0–3 scale (with 0 being not present and 3 being very salient) by two researchers. These two ratings were then averaged, and the pictures were randomized to different blocks to ensure an even distribution of difficulty throughout the procedure. The criteria rated were: (1) saliency of written language (Cerf et al., 2009); (2) saliency of road direction rule; (3) saliency of hidden object rule; and (4) apparent temperature. All pictures were standardized to be 1,670 pixels wide and between 600 and 750 pixels tall.

### tDCS

Subjects were randomized to receive anodal, cathodal, or sham stimulation over the left vlPFC. In each case, the return electrode was placed on the contralateral triceps. TDCS was administered by an ActivaDoseII Iontophoresis unit. Using a double-blind design, two of these units were connected to a blinding box, with 1 unit set to deliver an active dose of 2.0 mA and the other set to deliver a sham dose of 0.1 mA. Subjects were randomized to a specific switch on the blinding box, with the experimenter implementing the protocol unaware of the dosages associated with each switch. Two saline-soaked Amrex A5 (5 × 5 cm) sponges served as the electrodes, and these were attached to the subject’s arm with adhesive Coban wrap and to the subject’s head with an Amrex Velcro strap. Stimulation lasted 30 min and began after the baseline block. At 0 and 4 min after the start of stimulation, subjects completed a sensation questionnaire asking them to rate the degree of itching, heat and tingling on a 0–10 Likert-type scale. Subjects were informed that sensations rated 7 or above would prompt the termination of stimulation and end the experiment. After the first 5 min of stimulation, subjects began the 1st training block, with stimulation ending at the end of the 3rd training block.

### Profile of Mood States

To explore possible interactions between self-reported affect and performance improvements during the categorization task, subjects completed the short form of the Profile of Mood States (POMS) before stimulation (Shacham, 1983; Grove and Prapavessis, 1992). The POMS includes seven unique subscales, tension, anger, fatigue, depression, esteem, vigor, and confusion. Subjects also completed the POMS at the end of the experimental visit to assess any possible affective changes induced by tDCS or the experimental task.

### Remote Associates Test (RAT)

Before the experimental task, subjects performed the Remote Associates Test (RAT), a measure of convergent creativity (Mednick, 1962; Bowden and Jung-Beeman, 2003b). In the RAT the subject is presented with three words and is told to produce the 4th word that connects to the three presented words. For instance, a subject might be given the three words, “skate, pick, cream” where the appropriate answer would be “ice.” This test consisted of 15 unique items.

### Attention Networks Task

As previous work has found improvements in the ANT following F10 anodal stimulation (Coffman et al., 2012), the current study implemented the ANT before the task to tie baseline ANT performance with task learning. The ANT (Fan et al., 2002) consists of a combination of the flanker and cued reaction time tasks, and yields scores corresponding to three attention networks, alerting, orienting, and executive control. The Orienting subscale is created by subtracting the average reaction time in trials where there is a spatial cue from trials in which no spatial cue is presented. Larger numbers for the Orienting subscale indicate a greater reaction time advantage when a spatial cue is included. The alerting subscale is created by subtracting reaction time on trials with a temporal cue from reaction time on trials without. The Executive subscale acts as a measure of inhibitory ability and is calculated by subtracting average response time on congruent flanker trials from average response time on incongruent flanker trials.

### Data Analysis

To explicate learning of rules 1 and 2 within the main task, subjects were characterized as learners of rule 1, rule 2, both rules 1 and 2, or neither based on their categorization accuracy in test blocks 3 and 4. A subject was classified as a rule 1 learner if there was less than a 5% chance of having achieved their level of accuracy in the repeated images by chance alone. The value associated with a 5% chance was calculated from the distribution of categorization accuracy at baseline, such that scores above 60.5% were regarded as above chance. Rule 2 learners were similarly classified based on their performance in hidden object stimuli in test blocks 3 and 4. Based on these criteria, 19 subjects were classified as rule 1 learners, 14 as rule 2 learners, and 21 subjects as learners of neither rule. No subject had categorization accuracy above 60.5% for both rules 1 and 2.

Multinomial logistic regression was used to model the effect of performance on measures associated with the IFG and learning of the different rules in the main task. The full model contained one categorical variable, stimulation condition, and three continuous variables, Orienting subscale from the ANT, number of correct responses on the RAT, and the Tension subscale from the POMS.

## Results

### Subjects

Six subjects out of 60 were excluded from the final analysis. Two of these were excluded due to technical issues during data collection. An additional three subjects, one in each experimental group, were excluded for insufficient task engagement. Subjects were regarded as having insufficient task engagement if three criteria were met: classification accuracy was not appreciably above chance (60.5%) in at least one of the blocks, average response time was less than 1 s, and the pattern of response was indicative of disengagement. A response pattern was deemed as indicative of disengagement if responses were unidirectional (consistent 1’s or 2’s) or if the pattern of response consistently alternated across responses (1, 2, 1, 2, 1, 2…). One subject receiving cathodal stimulation reported a metallic taste and chose to leave the study during the first 5 min of stimulation. This left 54 subjects in the final analysis, equally distributed with 18 in each stimulation group. Rule group differences by demographic data and stimulation group are presented in Table 1.

**Table 1:** Rule group membership by stimulation group and demographics.

	Total	Anodal	Cathodal	Sham	Age			Male	Female
Rule	*N*	*N*	*N*	*N*	*Mean*	*SD*	*Range*	*N*	*N*
Rule 1	19	8	8	3	21.92	5.34	21	12	7
Rule 2	14	8	4	2	20.24	3.41	13	5	9
No Rule	21	2	6	13	26.34	11.53	38	6	15
Total	54	18	18	18	23.20	8.34	38	23	31

*Chi-square test of independence for stimulation condition $\chi_{\left(4,N=54\right)}^{2}$ = 15.49, *p* = 0.004. No significant demographic differences observed between rule groups*.

### Effects of tDCS on Category Learning

A mixed-model was run to explore the effect of stimulation conditions on overall learning (rules 1 and 2 combined through test block 2), the results of which are reported in detail elsewhere (Gibson et al., 2020). Both anodal and cathodal stimulation groups had significantly better improvement in categorization accuracy in comparison to sham, with anodal tDCS increasing categorization accuracy by 20.6% (SD = 16.1%) for an effect size of *d* = 1.71, and cathodal tDCS increasing categorization accuracy by 14.4% (SD = 11.8%) for an effect size of *d* = 1.16. In comparison, improvement in the sham stimulation group increased by 4.2% (SD = 11.7%) from baseline to test blocks 1 and 2.

### Multinomial Logistic Regression

Interaction terms were created for the three continuous variables and dummy coded stimulation conditions. Continuous variables were mean-centered before the creation of interaction terms (Kraemer and Blasey, 2004). None of these interaction terms reached significance, so they were removed from the model. Means and standard deviations of continuous variables between rule groups are presented in Table 2. Categorization accuracy for the respective rule groups across the experimental task is presented in (Figure 3).

**Table 2:** Means and standard deviations for continuous variables by rule learning group.

	Orienting		RAT # Correct		Tension	
Rule	*Mean*	*SD*	*Mean*	*SD*	*Mean*	*SD*
Rule 1	29.09	22.65	7.11	3.23	1.16	1.50
Rule 2	44.19	39.19	7.64	2.95	5.14	3.92
No rule	55.35	37.83	7.05	2.59	2.52	2.21

*Orienting score in milliseconds. Number correct on Remote Associates Test out of 23. Possible scores on the Tension subscale are between 0 and 24*.

Given the variables included in the model, three criteria were found to significantly predict subjects being categorized as rule 1 as opposed to those who learned neither rule. Receiving anodal stimulation made it 97.5% more likely that a subject would belong to the rule 1 group rather than the no rule group (OR = 0.025, 95% CI = 0.002, 0.328). Two of the three continuous variables, orienting score (OR = 1.051, 95% CI = 1.014, 1.09) and tension sub-score (OR = 2.165, 95% CI = 1.144, 4.097), were significant predictors of belonging to the no rule learned group as opposed to rule 1 learners. The same two continuous variables (orienting score (OR = 1.041, 95% CI = 1.004, 1.079) and tension sub-score (OR = 2.928, 95% CI = 1.503, 5.702) were also significant predictors of belonging to the rule 2 group as opposed to the rule 1 group. Betas and odds ratios for both comparisons are presented in Table 3. The model had an overall classification accuracy of 74.1%, ranging from 64.3% accuracy for classifying learners of rule 2, 78.9% for learners of rule 1, and 76.2% for no rule learners.

**Table 3:** Predictors of rule learning in multinomial logistic regression.

	No rule vs. rule 1 (reference)		Rule 2 vs. rule 1 (reference)	
Variable	B (SE)	OR (95% CI)	B (SE)	OR (95% CI)
Orienting	0.050 (0.018)*	1.051 (1.014, 1.090)	0.040 (0.018)*	1.041 (1.004, 1.079)
RAT # correct	0.251 (0.162)	1.285 (0.935, 1.766)	0.295 (0.018)	1.344 (0.941, 1.918)
Tension	0.773 (0.325)*	2.165 (1.144, 4.097)	1.074 (0.340)*	2.928 (1.503, 5.702)
Cathodal stim	-1.778 (1.023)	0.169 (0.023, 1.256)	-0.395 (1.389)	0.673 (0.044, 10.237)
Anodal stim	-3.688 (1.313)*	0.025 (0.002, 0.328)	-0.172 (1.445)	0.842 (0.050, 14.289)

*p < 0.05.

## Discussion

The results of this study support the interpretation that anodal tDCS over the rVLPFC is associated with an increased ability to learn rule 1, which requires an insight regarding the importance of traffic direction (Gibson et al., 2020). The results of the multinomial logistic regression additionally indicate that the quality of the attention subjects had as they began the task also selectively influenced category learning. Subjects who learned rule 2 featuring hidden objects had the largest tension sub-scores before stimulation at 5.14, compared to 1.16 in subjects who learned rule 1 featuring street directions. Rule 2 learners also had a gain in reaction time after receiving a spatial cue (Orienting) in the ANT, a difference of 44 ms compared to 29 ms in rule 1 learners. As predicted by Attentional Control Theory, the attention differences captured by the orienting subscale might themselves be the result of differences in state anxiety.

Differences in orienting driven by state anxiety possibly influenced subject learning during the experimental task. Rule 2 learners demonstrated a stronger reflexive saccade towards the positional cue in the ANT. Subsequently, rule 2 learners were more likely influenced by stimuli within the pictures presented during the experimental task, rather than by top-down goals (Schieber and Gilland, 2008; Allsop and Gray, 2014). Anxiety might have also disrupted working memory updating in rule 2 learners, further hindering systematic hypothesis testing (Eysenck and Calvo, 1992; Friedman and Miyake, 2004; Eysenck et al., 2007). In contrast, the lower relative anxiety of rule 1 learners allowed them to better control their attention, perhaps giving them an advantage in explicitly testing possible rules. Adding more complexity to an interpretation of the present results due to possible interactions with stimulation in the current study, changes in the relative activity of these attention systems are also associated with altered functioning of the prefrontal (Bishop, 2009; Eysenck and Derakshan, 2011) and ventrolateral prefrontal cortices (Ettinger et al., 2008; Fales et al., 2008).

Differences in convergent creativity, as measured by the RAT, may have also correlated with state anxiety, as previous research has shown that performing a convergent creativity task like the RAT is associated with decreases in mood (Chermahini and Hommel, 2012) and that this relationship is reciprocal (Bar, 2009), such that mood affects subsequent convergent creativity performance. Thus the same anxiety that promoted rule 2 learning could have also facilitated performance on the RAT. This may have also interacted with stimulation in the current study, as convergent creativity is associated with activity in the right hemisphere (Cerruti and Schlaug, 2009; Shah et al., 2013; Benedek et al., 2014; Hertenstein et al., 2019), and more subjects receiving anodal stimulation, rather than cathodal, gravitated towards rule 2 (8 vs. 4), while the number of rule 1 learners was even (8 vs. 8) between the anodal and cathodal groups, $\chi_{\left(4,N=54\right)}^{2}$ = 15.49, *p* = 0.004.

One debate between theoretical accounts of top-down and bottom-up processing involves the explanation of faster responses over time in stimuli that feature a single feature (Hillstrom, 2000; Wolfe et al., 2003; Wang et al., 2005; Awh et al., 2012). Do these faster reaction times indicate top-down processes (i.e., a tilde has been seen previously so attention is now purposefully being directed towards them), or do they indicate an unconscious priming effect that accrues over trials? The findings from the current study would seem to support top-down processes as 17 of 19 rule 1 learners mentioned street or traffic direction, and 12 of 14 rule 2 learners mentioned hidden objects as their main categorization criteria in a post-task debriefing. Subjects with higher baseline anxiety might have been more susceptible to an implicit orienting effect initially, but became fully aware of their search target as the training progressed. This awareness may have happened until later, however, in comparison to rule 1, learners are predisposed to top-down attentional control at the beginning of the task. While rule 1 learners appeared to identify rule 1 and begin improving in the first training block, as a group rule 2 learners did not begin to improve until the second training block. Subjects classified as no rule learners most often reported using architecture and written signage as categorization criteria.

Attentional control theory additionally posits that the detrimental effects of anxiety on performance can be overcome by compensatory strategies, with the employment of these strategies contingent upon motivation. In turn, motivation is thought to depend upon the clarity of task goals (Eysenck and Derakshan, 2011). In situations where goals are undefined, motivation and the use of compensatory strategies are likely to be low, leaving anxious individuals to fall back on an implicit attention system. This was demonstrated in what, to our knowledge, is the only other study to look at the effects of anxiety on category learning, where high anxiety was only detrimental to categorization performance when motivation was low (Hayes et al., 2009).

### Limitations

While the results of the multinomial logistic regression speak to the first rule subjects gravitate towards (rule 1 vs. rule 2), they do not provide an answer to why no subjects learned more than 1 rule. Research has conceptualized this phenomenon as the *satisfaction of search*, originally defined in radiology, where the successful detection of 2nd specific target drastically decreases after identification of the first (Tuddenham, 1962). This effect is exacerbated by time constraints (Fleck et al., 2010), as were present in the current study. Future use of these stimuli should attempt to define the parameters necessary for learning multiple rules.

Additionally, while none of the variables included in the model had statistically significant interactions with stimulation conditions, the presence of tDCS is still a caveat for interpretation that bears on any implications these findings have for theories of attention. Attentional control measured by antisaccade performance has shown that activation of the right VLPFC predicts antisaccade control (Ettinger et al., 2008), and contrastingly, orienting also demonstrates a right hemisphere bias (Corbetta et al., 2000; Fan et al., 2005). Thus, stimulation of this area might have prompted these processes to work against each other or interact in an unknown way. Complex tDCS-mediated effects could have also occurred in other parts of the brain, as the current introduced by tDCS is not confined to the area underneath the electrode (Spreng et al., 2012; Fonteneau et al., 2018). It cannot be ruled out, and may even be likely, that trepidation about tDCS itself was the driving force behind individual differences in state anxiety and not preexisting differences. Several decades of recent research have established the safety of tDCS (Bikson et al., 2016; Nikolin et al., 2018), but the placement of electrodes on the head is still a novelty for most subjects, and some apprehension about the procedure is possible despite best practices in informed consent. While rule 2 learners in the current study had tension sub-scores that were typical in a college-aged population (Shacham, 1983; Nyenhuis et al., 1999), the influence of this baseline anxiety is a question that should be addressed by future tDCS studies. A cross-over design would have allowed for an exploration of anxiety, separating out those who were only anxious before receiving their first ever tDCS dose from those with preexisting differences in anxiety, but the stimuli used in the current study precluded this design as exposure to a second or third iteration of the learning task would not be comparable to the first. A final limitation is the novelty of the learning task used. Further validation of the ability of this task to differentiate rule learning based on self-reported anxiety is needed.

## Conclusion

The current investigation demonstrates that individual differences can predict the trajectory of attention and that this trajectory in turn influences learning. While exploratory, these results fit within the structure of existing theories of attention and provide further evidence for the role anxiety plays within these theories. Importantly, these findings are relevant to real-world tasks that require effective orienting towards relevant stimuli, such as visual diagnosis, and piloting a car or plane, all of which can occur in situations of elevated anxiety or fatigue (Wilson et al., 2006; Allsop and Gray, 2014; Vine et al., 2016; Waite et al., 2019).

## Data Availability Statement

The datasets generated for this study are available on request to the corresponding author.

## Ethics Statement

The studies involving human participants were reviewed and approved by the U.S. Army Research Laboratory’s Human Research Protection Program, and by Advarra and Chesapeake Institutional Review Boards. The patients/participants provided their written informed consent to participate in this study.

## Author Contributions

BG and VC: conceptualization and methodology. BG and TM: software. BG and MH: formal analysis. BG: investigation and writing—original draft preparation. JH, AY and VC: resources. All authors: writing—review and editing. BG and VC: supervision. VC: project administration and funding acquisition. All authors contributed to the article and approved the submitted version.

## Conflict of Interest

VC is a Scientific Advisor of NeuroGeneces LLC. The remaining authors declare that the research was conducted in the absence of any commercial or financial relationships that could be construed as a potential conflict of interest.

## Footnotes

^1^http://www.maps.google.com
